# A genomic framework for tracking antibiotic resistance genes: global dissemination of *cfr*-family genes

**DOI:** 10.1128/aac.00122-26

**Published:** 2026-07-10

**Authors:** Hao Liu, Xianyang Huang, Xiaoqian Li, Yue Li, Zerui Shang, Huimin Zhou, Yifan Liu, Ge Yan, Jianjun Dai, Wan-Ting He

**Affiliations:** 1 Infectious Disease Prevention and Control Center, School of Pharmacy, China Pharmaceutical University 56651, Nanjing, China; 2 School of Life Science and Technology, China Pharmaceutical University, Nanjing, China; The Peter Doherty Institute for Infection and Immunity, Melbourne, Victoria, Australia

**Keywords:** antimicrobial resistance, mobile genetic element, epidemiology, evolutionary, *cfr*-like genes

## Abstract

The global spread of antimicrobial resistance (AMR) calls for advanced surveillance frameworks capable of tracking high-risk resistance genes across genomic, temporal, and ecological environments. To address this, we designed an integrated genomic epidemiology workflow, encompassing antibiotic resistance gene sequence alignment and phylogenetic classification, integration of whole-genome functional annotation, and individual analysis of key bacterial strains. Applying this framework, we obtained 1,026 chloramphenicol-florfenicol resistance (*cfr*)-like sequences through homology searches, and phylogenetic analysis resolved them into 11 monophyletic clusters, including the newly identified *cfr*(F) and *cfr*(G) variants. From the screening of 702,252 bacterial genomes, we identified 5,901 *cfr*-positive isolates comprising 11 distinct *cfr* variants. The earliest variant, *cfr*(G), was detected in a soil isolate dating to 1893. Genomic localization analysis revealed that *clbA*, *clbC*, *cfr*(E), *cfr*(F), and *cfr*(C) were putatively predominantly chromosomal, whereas *cfr*(G), *cfr*(B), *cipA*, and *clbB* were putatively mainly plasmid-borne. Importantly, *cfr-*like genes frequently co-localize with other last-resort antibiotic resistance genes (ARGs), including *vanA*, *optrA*, and carbapenemase genes, on the same isolate, promoting multidrug resistance. Beyond gene detection, this study clarifies the context-dependent transmission risks of resistance determinants and provides a strategic framework for anticipating resistance convergence and informing preemptive AMR surveillance.

## INTRODUCTION

Whole-genome sequencing (WGS) is increasingly prevalent in the detection and surveillance of drug-resistant bacteria. This technology has enhanced our understanding of the genomic diversity of these pathogens and enables global monitoring and antimicrobial resistance (AMR) tracking (1, 2). A substantial volume of such data is publicly shared, offering novel insights into the global distribution, diversity, and transmission dynamics of drug-resistant bacteria and their resistance genes (3–5).

Despite these advances in data generation, current research on antibiotic resistance genes (ARGs) remains predominantly focused on two approaches: comprehensive environmental resistome profiling through metagenomics and in-depth characterization in individual bacterial species using WGS (6–10). Significant limitations persist because existing public databases, although they catalog extensive data, often serve primarily as metagenomic annotation tools with limited gene-centric functionalities (11, 12). Although metagenomic surveys have revealed cross-species horizontal transfer of ARGs (13, 14), systematic analyses of the distribution of ARGs across different hosts remain critically underdeveloped (15, 16). Crucially, the lack of universally applicable gene-strain-environment transmission-tracing frameworks obstructs the elucidation of adaptive evolutionary pathways (17). Even in closed and controlled environments, such as bioregenerative life support systems, ARGs can persist and should be monitored (18), further underscoring the need for robust surveillance frameworks.

Linezolid, a synthetic oxazolidinone antibiotic approved in 2000, uniquely inhibits bacterial protein synthesis by suppressing the formation of the 70S initiation complex and blocking mRNA binding to the ribosome, a mechanism that minimizes cross-resistance (19–22). However, resistance genes, such as chloramphenicol-florfenicol resistance (*cfr*), have been identified, which encode a methyltransferase that modifies the A2503 site of the ribosome, conferring resistance to six classes of antibiotics: phenicols, lincosamides, oxazolidinones, pleuromutilins, streptogramin A, and tetracyclines (21, 22).

The evolutionary origin of the *cfr*-like genes remains unclear. It was first reported in *Staphylococcus* species but has since been detected in a range of other bacteria, including members of the genera *Enterococcus* and *Bacillus,* and species such as *Proteus vulgaris*, *Escherichia coli*, *Macrococcus caseolyticus*, and *Streptococcus suis*, and even in members of the phylum *Firmicutes* (21, 23–25), suggesting cross-species transmission potential (26). The *cfr* gene is typically plasmid-borne, although a novel variant was discovered within a thiamphenicol-resistant transposon, Tn*558*, in *Staphylococcus* isolates, and this variant includes an additional resistance gene region integrated into the *tnpC* reading frame, with evidence of transposition mediated by the IS*21-558* element (27). Further studies have revealed diverse genetic environments surrounding the *cfr* gene, including insertion sequences, such as IS*21-558*, IS*256*, IS*257*, and IS*1216E*, along with other associated resistance determinants (28, 29). Chromosomal integration of *cfr* is often flanked by insertion sequences, such as IS*256* and IS*Enfa5*, facilitating its mobilization through IS-mediated recombination (28). Recent analyses have documented 20 distinct genetic contexts for the *cfr* gene, with only three lacking insertion elements or transposon-related sequences (29). This structural variability underscores the gene’s potential for horizontal transfer across Gram-positive and Gram-negative bacteria (29).

Currently, the reported *cfr* variants are classified into nine types, namely *cfr*(A), *cfr*(B), *cfr*(C), *cfr*(D), *cfr*(E), *cipA*, *clbA*, *clbB*, and *clbC* (30–35). Specifically, *cfr*(A) was first identified on plasmid pSCFS1 in *Staphylococcus sciuri* (30), and its homologs naturally occur in *Bacillales* where they confer PhLOPS_A_ resistance via A2503 methylation (36). *cfr*(B) was detected in clinical *Enterococcus faecium* with two chromosomal copies embedded in a Tn*6218* transposon (37). *cfr*(C) resides on a putative 24-kb transposon in *Clostridium difficile* that forms a circular intermediate, indicating horizontal transfer potential (38). *cfr*(D) is located on a 103-kb plasmid flanked by IS*1216* elements in *E. faecium* (39). *cfr*(E), found in *C. difficile* from Latin America, shares only 51%–58% identity with known Cfr protein and resides within Tn*6218*-like transposons or ICEs (40). The remaining variants *cipA*, *clbA*, *clbB*, and *clbC* are less frequently reported and are mainly confined to *Bacillales* (36, 41).

**Fig 1:**
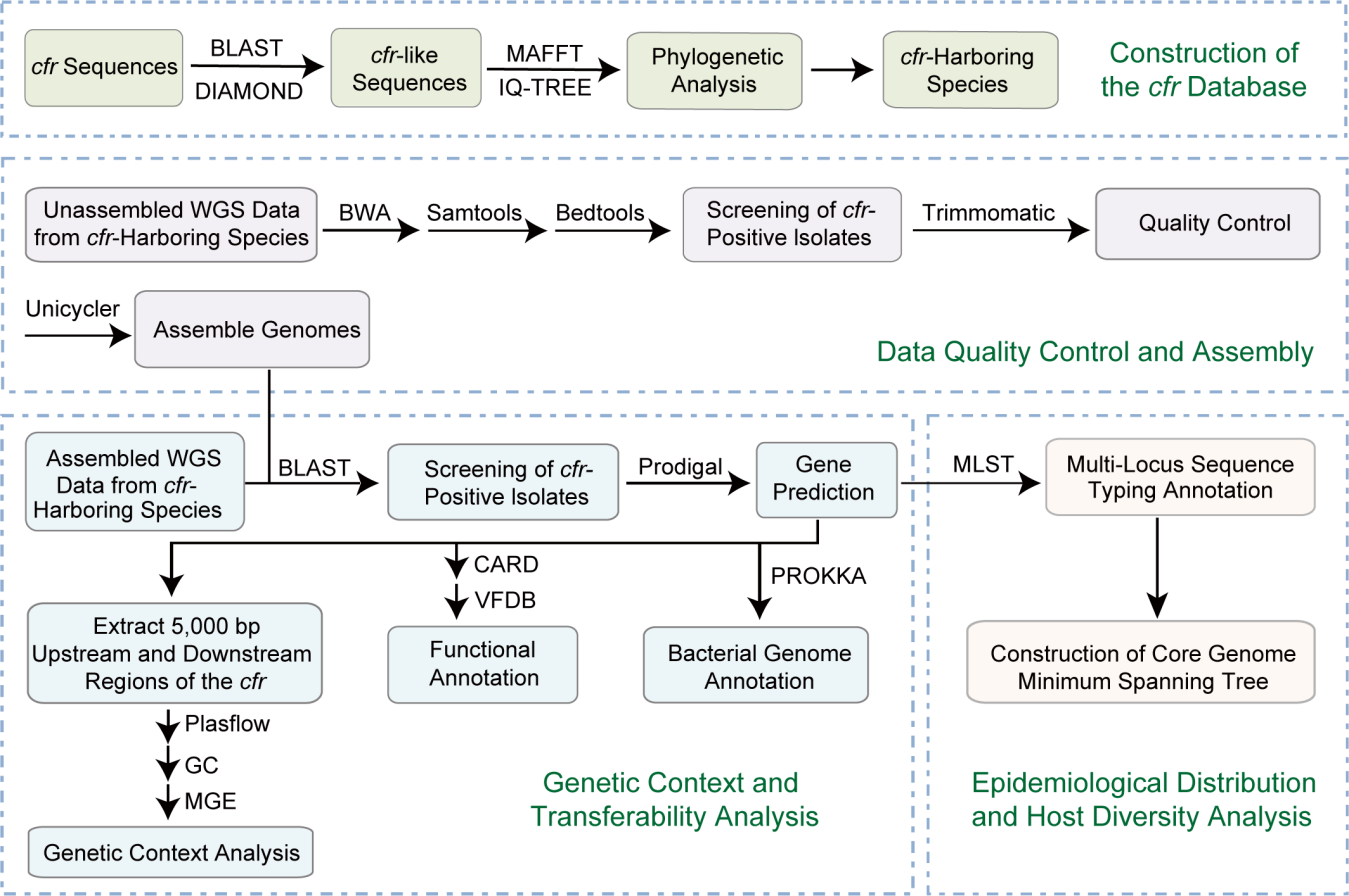
Flowchart of the analysis framework. Blue boxes represent each step of the operation, with arrows indicating the processes used. The blue dashed lines enclose the four major steps of the entire workflow: database construction and analysis of phylogenetic relationships and evolutionary history, analysis of unassembled genomes, analysis of assembled genomes, and analysis of epidemiological distribution characteristics and host diversity.

Building on these observations, we have designed a novel computational framework. The framework first establishes specialized databases for specific ARGs and employs maximum-likelihood methods to perform phylogenetic analysis and validation of the target genes, in order to clarify the evolutionary relationships among different clusters; it subsequently identifies positive strains from WGS data to investigate the gene’s distribution patterns across bacterial populations and to elucidate its transmission pathways (Fig. 1). Finally, we validate the utility and reliability of this framework through a case study of the *cfr* gene, and we investigate the biological origins of the *cfr* gene and the presence of naturally resistant bacteria. We also explore the gene’s distribution across bacterial populations, analyze its transmission pathways, and identify key factors driving the spread of resistant bacteria and genes.

## RESULTS

### Identification and phylogenetic classification of *cfr*-like sequences

**Fig 2:**
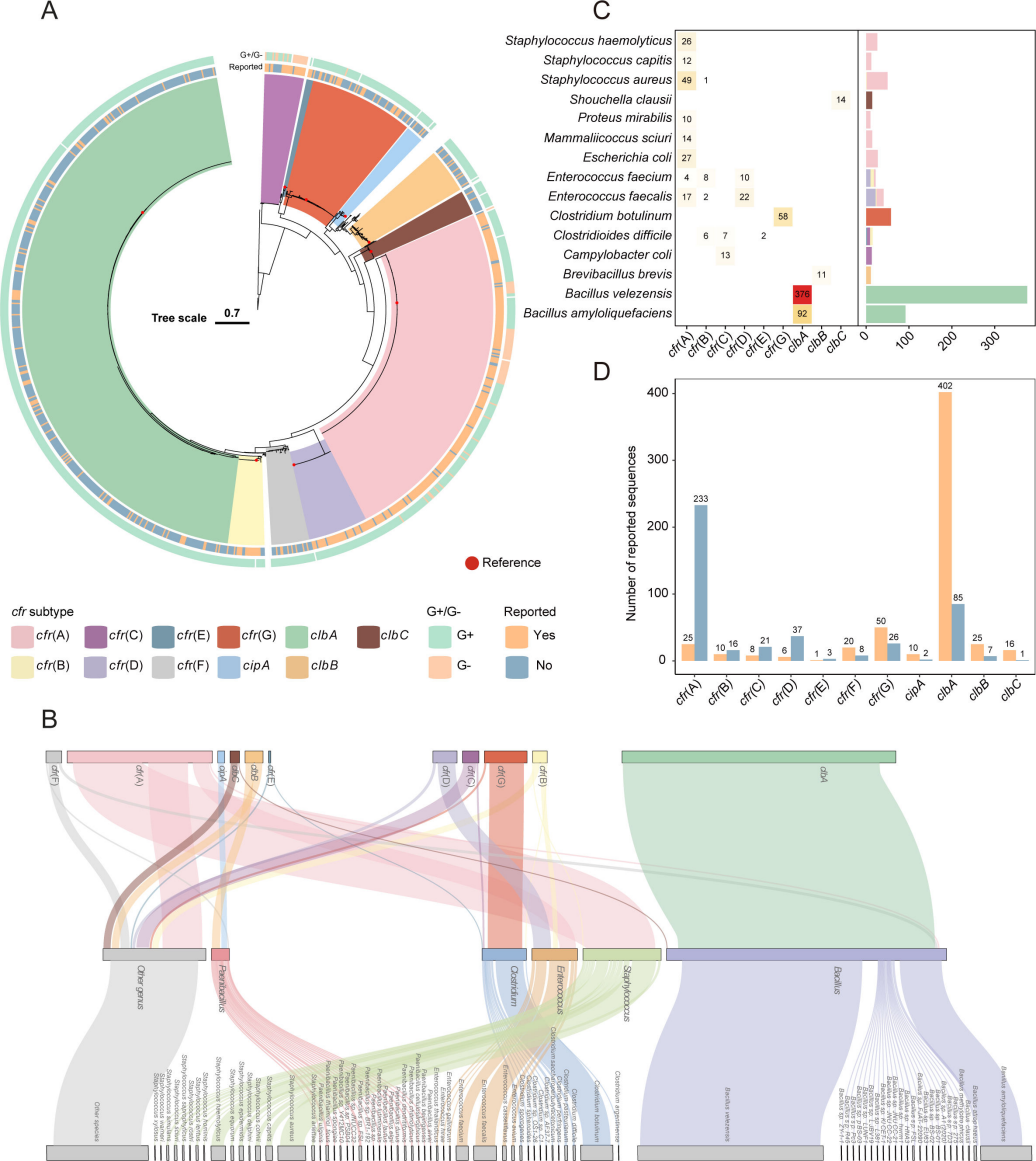
Diversity, taxonomic distribution, and phylogenetic relationships of *cfr* and *cfr*-like genes. (**A**) Phylogenetic tree of all *cfr* genes. The phylogenetic tree was estimated using a maximum likelihood method based on *cfr* nucleotide sequences. The tree was midpoint-rooted for clarity only, and the scale bar represents the number of nucleotide substitutions per site. Red dots indicate reference sequences. The two outer rings indicate reporting status (orange, previously reported; blue, newly discovered) and host Gram-staining property (green, Gram-positive; orange, Gram-negative), respectively. Inner ring colors represent different *cfr* subtypes. (**B**) Sankey diagram of *cfr* subtypes at the genus and species level across different taxa. (**C**) Distribution of *cfr* gene types and their counts in the top 15 species with the highest number of *cfr* genes in the database. The Y-axis represents species, and the X-axis shows the subtypes and corresponding counts of *cfr* genes carried by each species. The figure is divided into two parts: the left side presents the specific counts of the *cfr* gene subtypes per species; the right side displays a stacked bar chart of the total *cfr* gene counts per species, with different colors representing distinct *cfr* gene subtypes and the stacked lengths within each bar corresponding to the counts of each subtype. (**D**) The *cfr* gene typing and expansion in the *cfr* and its homologs gene database. Orange represents previously reported sequences, while blue indicates newly discovered *cfr*-like sequences. The labels on the X-axis represent the *cfr* subtypes in the database, and the Y-axis represents the number of each *cfr* subtype in the database.

Here, based on homology searches against the National Center for Biotechnology Information (NCBI) Nucleotide Database (NT) and Non-Redundant Protein Sequence Database, a total of 1,026 *cfr*-like sequences with amino acid identity >50% were obtained in this study. The phylogenetic analysis revealed that *cfr* and its variants were resolved into 11 distinct clusters (Fig. 2A). In addition to the previously reported *cfr*(A), *clbA*, *clbC*, *cfr*(B), *clbB*, *cipA*, *cfr*(C), *cfr*(D), and *cfr*(E), the novel *cfr*-like genes identified in this study were tentatively designated as *cfr*(F) and *cfr*(G), respectively, in accordance with the macrolide-lincosamide-streptogramin B (MLS_B_) nomenclature system maintained by Marilyn Roberts (http://faculty.washington.edu/marilynr/) to ensure consistency. The amino acid sequence identity between *cfr*(G) and the other nine known variants ranges from 51.94% to 62.06%. *cfr*(F) shares 82.32%, 81.74%, and 76.25% amino acid identity with *clbA*, *cfr*(B), and *cfr*(A), respectively, and between 52.96% and 65.22% with the remaining variants. The novel genotype *cfr*(F) consistently formed a distinct monophyletic cluster, clearly separated from other *cfr*-like genotypes, and exhibited the closest phylogenetic affinity with *cfr*(D) and *cfr*(B). Meanwhile, the novel genotype *cfr*(G) displayed the closest relationship with *cfr*(E).

The currently identified *cfr*-like genes comprise 11 variants. Among these, the *clbA* variant was the most prevalent, accounting for 47.6% (488/1,026) of the total sequences (Fig. 2B). The top five bacteria at the genus level contributing to the *cfr*-like sequences were: *Bacillus* (48.2%, 498/1,026), *Staphylococcus* (13.5%, 139/1,026), *Enterococcus* (7.9%, 81/1,026), *Clostridium* (7.7%, 79/1,026), and *Paenibacillus* (4.0%, 41/1,026). The most common source was *Bacillus velezensis* (36.6%, 376/1,026) (Fig. 2C). A total of 438 *cfr*-like sequences were previously reported, while the majority (588 sequences) represent newly identified variants according to our framework. Among these, *clbA* accounted for the largest number of newly discovered variants (402 sequences) (Fig. 2D).

### Epidemiological overview and genomic localization of *cfr*-positive isolates

**Fig 3:**
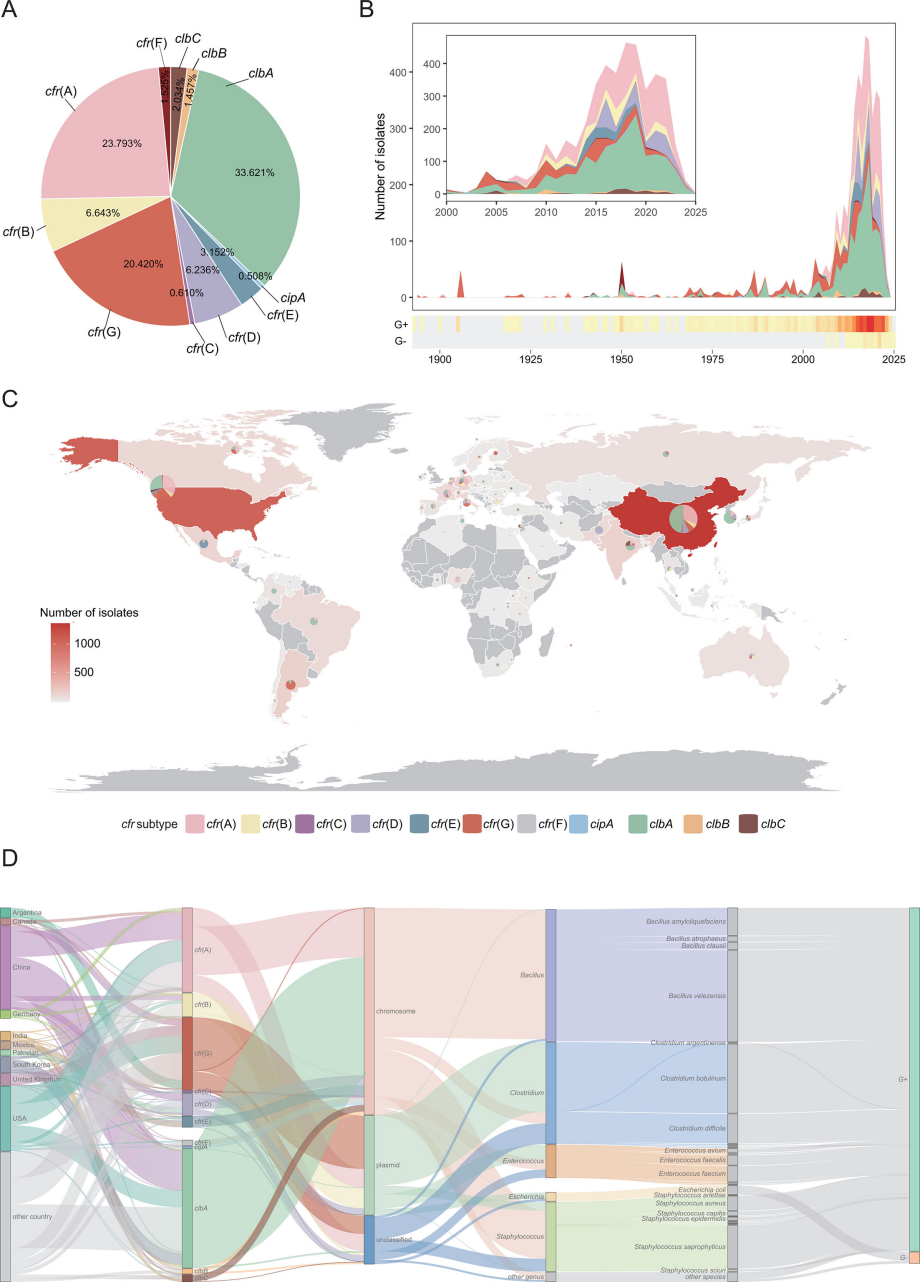
Distribution of bacterial strains for genomic analysis. (**A**) Composition of *cfr* gene subtypes. The pie chart illustrates the proportional distribution of different *cfr* subtypes in the data set. The size of each sector represents the relative abundance of the corresponding *cfr* subtype. (**B**) Temporal distribution of different *cfr* subtypes. The main panel shows the full timeline, while the inset in the upper left corner provides a magnified view of the period after 2000. The X-axis represents time (year), and the Y-axis indicates the frequency of each subtype. The bar plot at the bottom displays the number of *cfr*-carrying Gram-positive and Gram-negative bacteria per year. (**C**) Global distribution of *cfr*-positive isolates. Countries with *cfr*-positive isolates are shown in a red gradient background, with darker shades indicating higher numbers of *cfr*-positive isolates. Gray background represents countries without detected *cfr*-positive isolates. (**D**) Sankey diagram depicting the distribution and flow of *cfr*-positive isolates across country of isolation, *cfr* variant, genomic localization, host genus, host species, and Gram-staining property.

From the large-scale WGS data set curated in this study, comprising 702,252 WGS entries, a total of 5,901 *cfr*-positive isolates were detected, encompassing all 11 *cfr* variants. The most prevalent variant was *clbA* (33.6%, 1,984/5,901), followed by *cfr*(A) (23.8%, 1,404/5,901) and *cfr*(G) (20.4%, 1,205/5,901); the remaining variants occurred at frequencies below 7% (Fig. 3A). Notably, the earliest *cfr*-positive samples were collected in 1893, consisting of two soil samples of *Clostridium pasteurianum* from Russia. The majority (82/90) of isolates collected between 1893 and 1935 carried *cfr*(G), suggesting that *cfr*(G) may represent the ancestral form of the gene. *clbA* emerged in 1940 and gradually became co-dominant with *cfr*(G), while *cfr*(A), *cfr*(B), and *cfr*(D) increased in frequency only after 2005 (Fig. 3B). Geographically, these isolates originated from 77 countries, with the highest proportion originating from China (23.6%, 1,391/5,901), followed by the United States (18.4%, 1,083/5,901), South Korea (4.6%, 271/5,901), and the United Kingdom (3.3%, 197/5,901) (Fig. 3C).

Genomic localization analysis revealed variant-specific and host-dependent patterns (Fig. 3D). *clbA*, *clbC*, *cfr*(E), *cfr*(F), and *cfr*(C) were predominantly or exclusively chromosomal, whereas *cfr*(G), *cfr*(B), *cipA*, and *clbB* were predominantly plasmid-borne. *cfr*(A) and *cfr*(D) exhibited mixed localization, with *cfr*(A) favoring the chromosome and *cfr*(D) showing a near-even distribution. At the genus level, *Bacillus* and *Paenibacillus* almost exclusively carried chromosomal variants (>99.0%, 2,153/2,155 and 86/86), consistent with clonal expansion. *Staphylococcus* harbored primarily chromosomal *cfr*(A) (85.1%, 804/945) with a minor plasmid-borne fraction. In contrast, *Clostridium* was dominated by plasmid-borne *cfr*(G) (73.6%, 884/1,201) and *cfr*(B) (98.9%, 269/272), while *Enterococcus* exhibited a more balanced mix of plasmid-borne *cfr*(D) (60.8%, 161/265) and *cfr*(B) (89.5%, 68/76) alongside chromosomal *cfr*(A) (94.4%, 67/71). *Escherichia* and *Proteus* carried *cfr*(A) almost exclusively on plasmids (≥99.0%, 102/103 and 6/6). A striking example of host influence was *cfr*(B), which was chromosomally integrated in *Enterococcus faecalis* yet remained predominantly plasmid-borne in *E. faecium*, underscoring the role of host genomic context in shaping variant localization.

### Chromosomal integration features and plasmid-associated mobile elements of *cfr* variants

**Fig 4:**
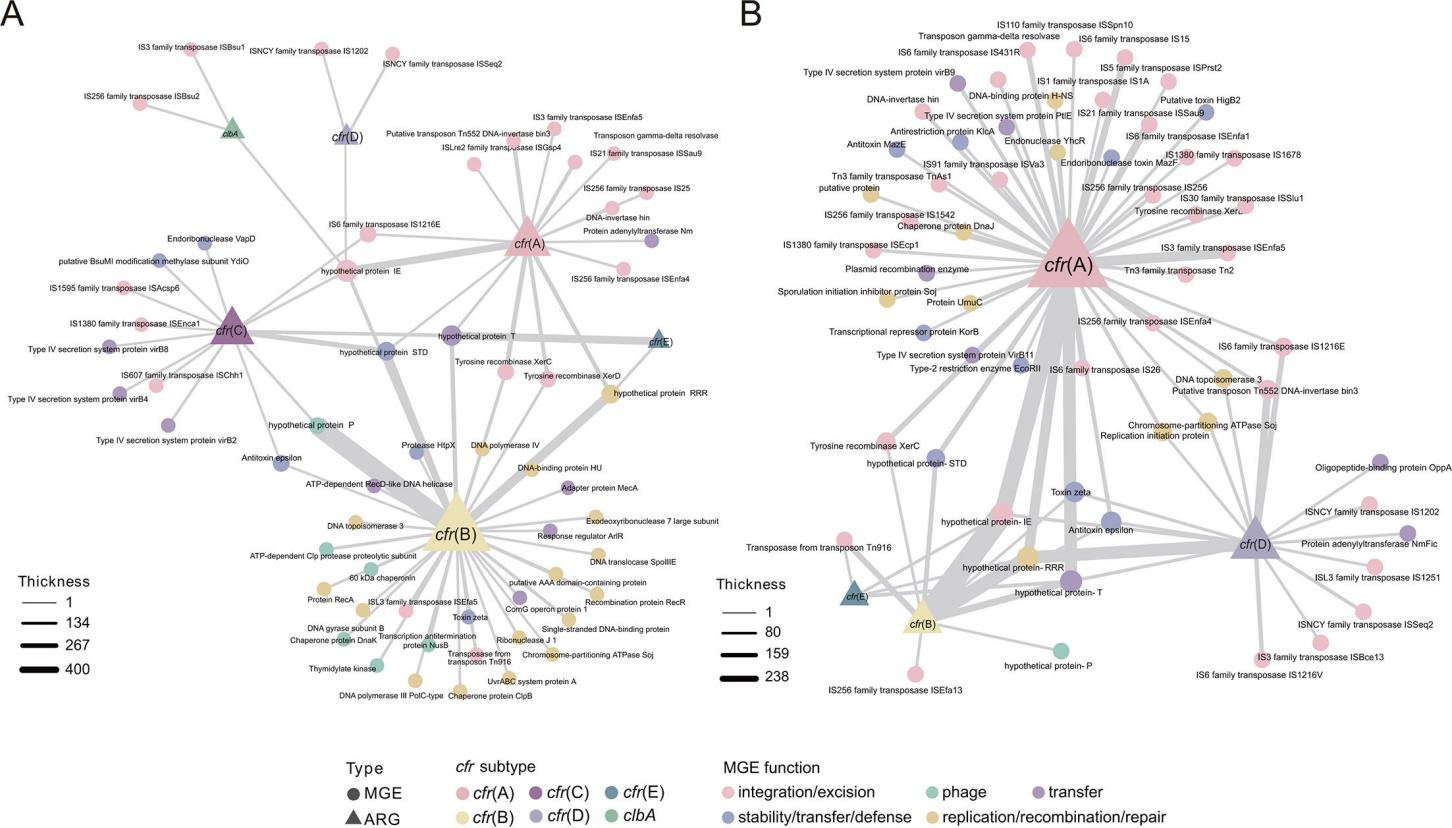
Genomic localization and insertion sequence carriage of the *cfr* gene. (**A**) Network diagram illustrating associations between chromosomal *cfr* genes and various mobile genetic elements. Triangular nodes represent *cfr* genes, circular nodes represent mobile genetic elements, and the thickness of the connecting lines reflects the frequency or strength of their associations. Different colors represent different subtypes. (**B**) Network diagram illustrating associations between plasmid-borne *cfr* genes and various mobile genetic elements. Triangular nodes represent *cfr* genes, circular nodes represent mobile genetic elements, and the thickness of the connecting lines reflects the frequency or strength of their associations. Different colors represent different subtypes.

When located on the chromosome, *cfr* variants exhibited distinct integration patterns indicative of stable genomic incorporation (Fig. 4A). *cfr*(B) displayed the most complex chromosomal context, with flanking regions enriched in core cellular function genes involved in DNA replication, recombination, and repair (340 cases), as well as abundant phage-related genes (539 cases), consistent with integration into a prophage or genomic island. *cfr*(A) chromosomal integrations were less frequent and primarily associated with integration/excision functions (384 cases) and replication genes (55 cases). *cfr*(E) was almost exclusively linked to transfer-related genes (157 of 167 cases), while *cfr*(C) exhibited a mixed profile, including stability, transfer, and defense genes (83 cases). *clbA* integrations were associated solely with integration/excision genes (17 cases) involving IS*256* or IS*3* family transposases, suggesting a simpler, transposase-mediated mechanism. *cfr*(D) showed only modest involvement with integration/excision (68 cases). In contrast, plasmid-borne *cfr* variants were consistently associated with diverse mobile genetic elements, notably transposases of the IS*3*, IS*6*, Tn*3*, and Tn*916* families, as well as type IV secretion system components and toxin-antitoxin modules, confirming their capacity for horizontal dissemination (Fig. 4B).

### Evidence of horizontal transfer based on GC anomalies

GC-content anomaly analysis identified regions with GC composition significantly deviating from the genomic background, suggestive of recent horizontal acquisition (Fig. S1A). For instance, in *Clostridium difficile* strain GCA_022281965 (GC 49.9%, deviation +2.80σ) carrying the *cfr*(E) variant, the flanking region of *cfr*(E) exhibited elevated GC content and contained the plasmid replication gene *repA* adjacent to the chromosomally located *cfr*(E), indicating a chromosomally integrated plasmid remnant. Similarly, in strain GCA_018893195 (GC 49.4%, deviation +2.69σ), multiple copies of mobA_2, a key conjugative transfer gene, were enriched near plasmid-borne *cfr*(E), supporting the presence of a conjugative plasmid or transposon. In contrast, GC-skew anomaly regions (Fig. S1B) lacked typical mobile genetic elements, suggesting that skew deviations likely reflect intrinsic sequence features or localized mutational pressure rather than recent horizontal transfer events.

### Prevalence of last-resort ARGs in *cfr*-positive isolates

**Fig 5:**
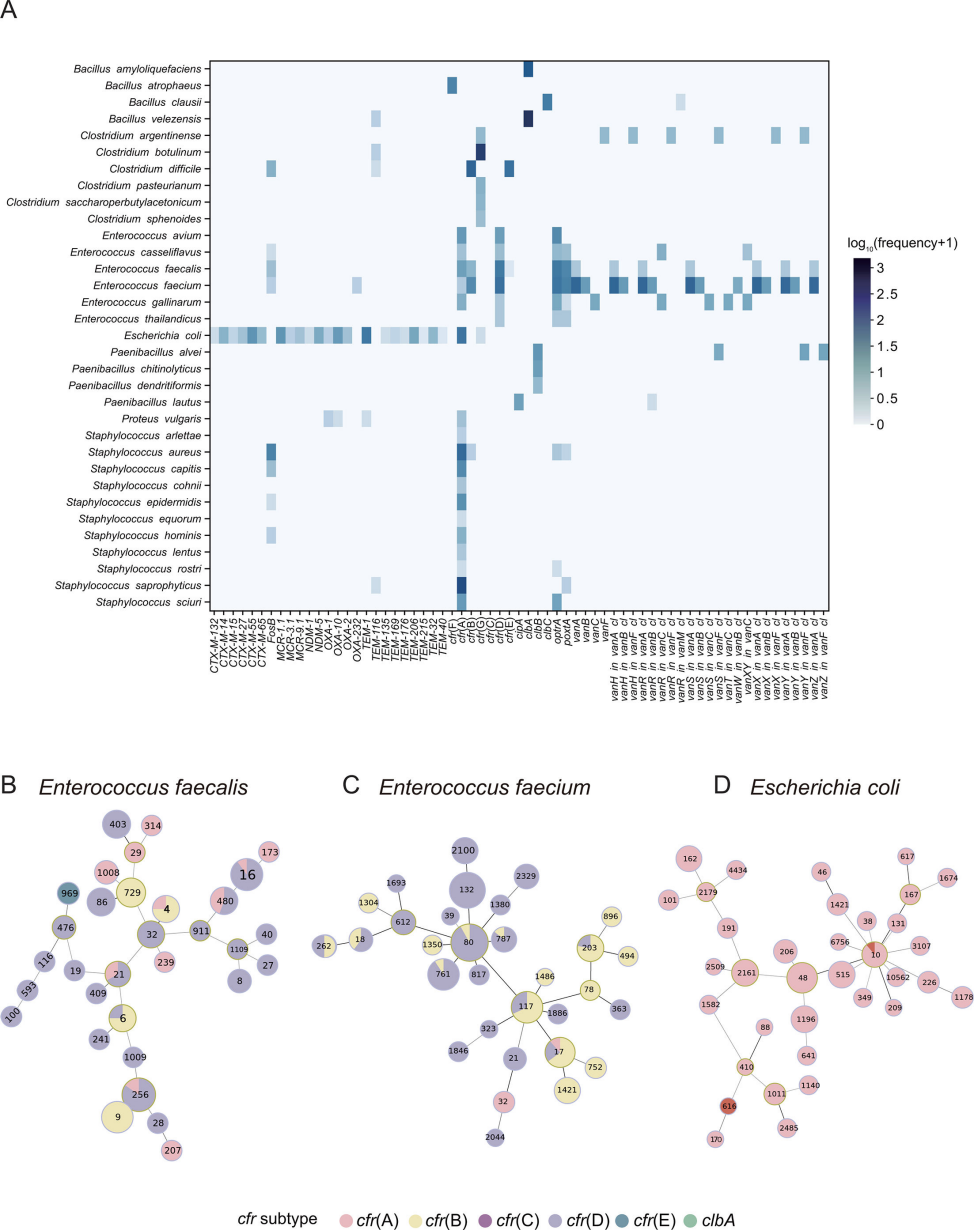
Analysis of the genetic environment of *cfr*. (**A**) Heatmap of last-resort antibiotic resistance genes in *cfr*-positive isolates. Distribution of last-resort antibiotic resistance genes across bacterial species. Heatmap shows log10-transformed gene frequencies after adding 1. (**B**) Minimum spanning tree of multilocus sequence type (MLST) for *cfr*-positive *Enterococcus faecalis*. Circles and numbers represent the different STs detected in this study. Different colors represent distinct *cfr* gene variants. (**C**) Minimum spanning tree of MLST for c*fr*-positive *Enterococcus faecium*. Circles and numbers represent the different STs detected in this study. Different colors represent distinct *cfr* gene variants. (**D**) Minimum spanning tree of MLST for c*fr*-positive *Escherichia coli*. Circles and numbers represent the different STs detected in this study. Different colors represent distinct *cfr* gene variants.

Analysis of the resistance gene profiles of *cfr*-positive bacterial isolates revealed the carriage of multiple last-resort ARGs (Fig. 5A). These included carbapenem resistance genes, the vancomycin resistance gene *van*, and the linezolid resistance gene *optrA*. The *cfr*-positive bacteria commonly exhibited a co-carriage pattern of multiple drug resistance genes. Among them, *cfr*-positive *Clostridium* and *Enterococcus* isolates frequently harbored *van* genes, with *Clostridium* primarily carrying the *vanF* gene cluster and *Enterococcus* predominantly carrying the *vanA* gene cluster. Within *Enterococcus*, *cfr*-positive isolates mainly harbored the *cfr*(A) and *cfr*(D) variants, and demonstrated a high detection rate of the *optrA* and *poxtA* genes, both of which confer resistance to linezolid. In contrast, *cfr*-positive *Bacillus* isolates primarily carried the *cfr*(A) variant and did not carry other last-resort ARGs.

Specifically, in *E. faecalis*, three instances of *vanA* clusters co-occurring with *cfr*(B) and two instances co-occurring with *cfr*(D) were detected. In *E. faecium*, 27 instances of *vanA* clusters co-occurring with *cfr*(B) and 184 instances co-occurring with *cfr*(D) were identified. Cases of *vanB*-type gene clusters co-occurring with *cfr* genes were also present. Furthermore, this study conducted a large-scale analysis of the co-occurrence of two oxazolidinone resistance genes, *cfr*(D) and *optrA*, in enterococci. Among 366 *cfr*(D)-positive enterococcal isolates, the co-occurrence rate of *optrA* varied by species: 100% in both *E. avium* and *E. gallinarum*, 80.5% in *E. faecalis*, 71.4% in *E. faecium*, and 50% in *E. casseliflavus*. In *cfr*-positive *E. coli*, a variety of resistance genes were detected, including extended-spectrum β-lactamase genes, carbapenemase genes, oxacillinase genes, multiple *bla*_TEM_ variants, and colistin resistance genes.

To further delineate genus-specific patterns, *Enterococcus* was examined as a clinically relevant model. *cfr*-positive *E. faecalis* isolates harbored four variants [*cfr*(A), *cfr*(B), *cfr*(D), *cfr*(E)] and were assigned to 32 known STs, with ST256 and ST16 predominating; six STs each carried two *cfr* variants (Fig. 5B). *E. faecium* isolates carried three variants [*cfr*(A), *cfr*(B), *cfr*(D)] across 31 STs, with ST80 and ST132 most prevalent; ST17 strains contained all three variants, and seven other STs carried two variants (Fig. 5C). In *E. coli*, only *cfr*(A) (141 isolates) and *cfr*(C) (2 isolates) were detected, distributed among 34 STs with ST48 as the dominant type. ST10 strains harbored both variants, whereas ST616 carried only *cfr*(C) (Fig. 5D), indicating that *cfr*(A) is the predominant variant in this species.

### Diversity and transmission features of *cfr* genes across key species

**Fig 6:**
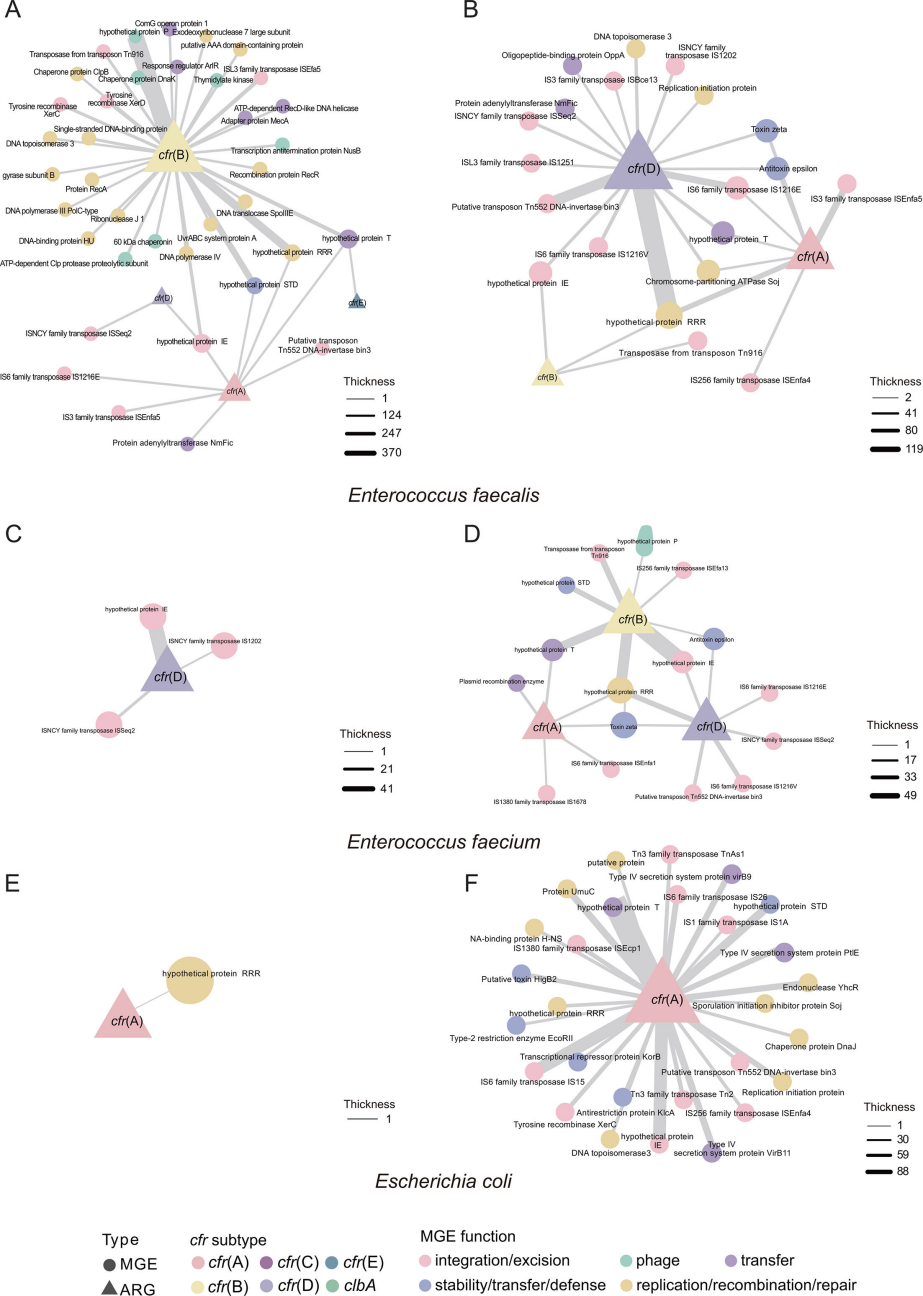
Population structure and genetic associations of *cfr*-positive *Enterococcus faecalis*, *Enterococcus faecium* and *Escherichia coli*. Triangular nodes represent *cfr* genes, circular nodes represent mobile genetic elements, and the thickness of the connecting lines reflects the frequency or strength of their associations. Different colors represent different subtypes. (**A**) Network diagram illustrating associations between *cfr* genes and mobile genetic elements across different chromosomes in *Enterococcus faecalis*. (**B**) Network diagram illustrating associations between *cfr* genes and mobile genetic elements across different plasmids in *Enterococcus faecalis*. (**C**) Network diagram illustrating associations between *cfr* genes and mobile genetic elements across different chromosomes in *Enterococcus faecium*. (**D**) Network diagram illustrating associations between *cfr* genes and mobile genetic elements across different plasmids in *Enterococcus faecium*. (**E**) Network diagram illustrating associations between *cfr* genes and mobile genetic elements across different chromosomes in *Escherichia coli*. (**F**) Network diagram illustrating associations between *cfr* genes and mobile genetic elements across different plasmids in *Escherichia coli*.

Further analysis revealed distinct colonization and dissemination strategies adopted by different *cfr* genes in *E. faecalis* (Fig. 6A and B). *cfr*(B) is almost exclusively localized to the chromosome. Its flanking regions exhibit a highly complex gene composition, encompassing genes involved in core cellular functions, such as DNA replication/repair and transcriptional regulation, suggesting stable integration into a chromosomal genomic island or prophage region. In contrast, *cfr*(D) is predominantly plasmid-borne, displaying the characteristic features of a typical mobile resistance module. *cfr*(A) on plasmids shows a significant and specific association with the IS*3* family transposase IS*Enfa5* within a relatively simple genetic context, indicating it may function primarily via transposition mediated by this specific IS element.

Compared to *E. faecalis*, both the detection frequency of *cfr* genes and the complexity of their genetic contexts are substantially lower in *E. faecium* (Fig. 6C and D). This difference is particularly striking for *cfr*(B). In *E. faecium*, *cfr*(B) is found primarily on plasmids, flanked by a homogeneous environment consisting almost entirely of hypothetical protein-coding genes. This stands in stark contrast to its complex and stable chromosomal context in *E. faecalis*, suggesting that in *E. faecium*, *cfr*(B) may not have undergone deep genomic integration and likely persists merely as a mobile element on plasmids.

In *E. coli*, chromosomal integration of *cfr*(A) was detected in only a single isolate, where the flanking region contained merely a hypothetical protein and a replication/recombination/repair gene, indicative of a recent and likely unstable acquisition event (Fig. 6E and F). Plasmid-borne *cfr*(A) was far more prevalent and resided within a complex mobile resistance platform harboring numerous transposase families (IS*1*, IS*1380*, IS*256*, IS*6*, Tn*3*, and IS*Enfa5*), type IV secretion system components, and toxin-antitoxin systems. Thus, *cfr*(A) dissemination in *E. coli* is predominantly plasmid-driven, with chromosomal integration being a rare and transient phenomenon.

## DISCUSSION

The clinical consequences of AMR are severe and escalating, with an estimated 4.71 million associated deaths globally in 2021 and over 39 million projected by 2050 (42). This mounting threat, underscored by the WHO Bacterial Priority Pathogens List (43), necessitates systematic profiling of resistance markers. Therefore, we established a multidimensional analytical framework (Fig. 1) that integrates dedicated database construction, sensitive homology screening (BWA and BLAST), and comprehensive characterization of resistance profiles, mobile genetic contexts, and host phylogenies.

Our comprehensive genomic analysis reveals a fundamental divergence in the genomic lifestyle of *cfr* variants. Some variants are predominantly chromosomally located, while others are mainly plasmid-borne. The identification of *cfr*(G) in samples dating back to 1893 supports its status as an early, potentially ancestral variant (38, 44). The subsequent rise of *clbA* (from ~1940) and the recent increase of *cfr*(A) and *cfr*(D) variants likely trace the escalating selection pressures of the antibiotic era. This trajectory, from environmental reservoir (44) to chromosomal adaptation and finally to mobile MDR plasmids, illustrates the adaptive pathway of a resistance determinant under intense anthropogenic selection. In the phylogenetic tree (Fig. 2A), *cfr* sequences from both Gram-positive and Gram-negative bacteria cluster together in several clades, suggesting that these genetic determinants may have repeatedly crossed the Gram-staining barrier. Notably, *cfr*(G) was detected in Gram-positive bacteria as early as 1893 (45), but did not appear in Gram-negative bacteria until 2021 (46), a temporal pattern that provides direct evidence for cross-barrier dissemination driven by antibiotic selective pressure.

In the chromosomally associated variants, *clbA*, *clbC*, *cfr*(E), *cfr*(F), and *cfr*(C) are predominantly or exclusively chromosomally integrated. In *Bacillales*, *clbA* and *clbC* exhibit almost exclusive chromosomal localization, contrasting with earlier reports that described *clbA* as plasmid-encoded (36), suggesting that they are likely stable components of the host genome. Given the constraints of short-read assembly, plasmid versus chromosomal assignments derived from PlasFlow are presumptive and may warrant validation by long-read sequencing or experimental approaches. Notably, *cfr*(C) sequences from the CARD database failed to cluster with the originally reported *cfr*(C) and were therefore designated as *cfr*(G). Subsequent localization analysis revealed that *cfr*(G) is nearly always plasmid-borne, whereas *cfr*(C) is predominantly chromosomal. This phylogenetic and ecological discrepancy indicates that the current *cfr*(C) classification may require re-evaluation and potential subdivision.

The plasmid-associated variants *cfr*(G), *cfr*(B), *cipA*, and *clbB* are predominantly plasmid-borne, while *cfr*(A) and *cfr*(D) display mixed localization patterns. *cfr*(A) is predominantly chromosomal in *Staphylococcus* and *Bacillus*, yet plasmid borne in *Enterococcus* and *Escherichia*, while *cfr*(D) is nearly evenly split with a slight plasmid preference in *Enterococcus*. The *cfr*(B) variant exhibits host-dependent localization: it is predominantly plasmid-borne in *E. faecium* and *Clostridium*, often associated with transposons, such as Tn*6218* (37, 47), but can be chromosomal in *E. faecalis*. Plasmid-borne *cfr*(A) is frequently associated with IS*Enfa5*, echoing the known mobility role of related IS*21-558* elements (28). Notably, *cfr*-positive isolates have been increasingly identified in diverse ecological niches, including livestock-associated *Staphylococcus aureus* CC398 from pig farms in Korea (48) and clinical *Enterococcus* isolates from Nigeria co-harboring multiple linezolid resistance determinants (49). Focusing on clinically critical enterococci, large-scale analysis of *cfr*(D)-positive isolates (*n* = 366) reveals high yet species-dependent co-occurrence rates with *optrA* (71.4% in *E. faecium*, 80.5% in *E. faecalis*). This contrasts with earlier small-scale studies reporting universal co-carriage (50, 51), indicating that the association is not absolute. This necessitates a paradigm shift in surveillance from detecting single genes to tracking high-risk co-resistance genotypes and their associated plasmids, exemplified by clinical reports of *optrA*, *cfr*(D), and *poxtA* coexisting in a single *E. faecium* isolate in Pakistan (52), and the co-localization of *optrA*, *cfr*(D), and *vanA* on a linear plasmid in a clinical *E. faecium* isolate exhibiting concurrent resistance to linezolid, vancomycin, and multiple other antibiotics (53).

In summary, this study established a multidimensional analytical framework that systematically integrates large-scale genomic screening with localization analysis, mobile element annotation, and host metadata. This framework enables the systematic expansion of known resistance gene variant repertoires, distinguishes between vertical inheritance and horizontal transmission strategies to inform diffusion risk assessment, and captures co-carriage patterns of last-resort resistance genes, providing a complete analytical pipeline from gene discovery to risk assessment for proactive AMR surveillance.

## MATERIALS AND METHODS

### Acquisition of *cfr* reference sequences

The workflow for data acquisition began with the retrieval of *cfr* reference sequences (Fig. 1). For comprehensive identification of potential *cfr* genes, the complete nucleotide sequences and their encoded protein sequences of all nine currently characterized *cfr* variants [*cfr*(A), *clbA*, *clbC*, *cfr*(B), *clbB*, *cipA, cfr*(C), *cfr*(D), and *cfr*(E)] were retrieved from CARD (11) (https://card.mcmaster.ca/) and GenBank as reference sequences. The reference sequences have been added to the Supplementary file (Table S1).

### Homologous sequence identification and database construction

To systematically identify sequences homologous to *cfr* and its orthologues, we established a search database based on the reference protein and nucleotide sequences of *cfr*. The NT was retrieved from NCBI (https://www.ncbi.nlm.nih.gov, updated as of 19 December 2024) and indexed for subsequent homology searches.

### Acquisition of bacterial genomes for *cfr* gene identification

To systematically identify bacterial strains harboring the *cfr* resistance genes, we retrieved from NCBI GenBank and SRA genome data for all *cfr*-like-positive species within the five genera showing the highest abundance of *cfr*-like sequences. Since these five genera are Gram-positive, three previously reported *cfr*-carrying Gram-negative species, namely *E. coli*, *Campylobacter coli*, and *Proteus vulgaris*, were also included to ensure Gram-negative representation. The downloaded genomes included both short-read sequencing data (primarily from Illumina HiSeq and NovaSeq platforms) and hybrid assembly data (Illumina + Nanopore). The final curated data set comprised 702,252 non-redundant WGS entries; these isolates were sourced from 155 countries and regions, with the majority originating from the United States (33.95%) and China (9.0%). Our analysis encompassed 36 species spanning eight clinically relevant genera. Among all samples, *E. coli* (64.38%) was the most prevalent, followed by *Staphylococcus aureus* (24.2%), *E. faecium* (7.4%), and *E. faecalis* (2.3%).

### Identification, classification, and phylogenetic validation of *cfr* homologs

Sequence alignments were performed using BLASTn v2.15.0 and BLASTx (54, 55), with DIAMOND v2.1.9.163 (56) employed to accelerate BLASTx computations. Rigorous filtering criteria were applied, including an E-value cutoff of 10^−5^, sequence identity >50%, and coverage >70%. For the obtained *cfr*-like sequences, hmmscan from the HMMER package v3.4 (57) was used to detect the presence of the CX₃CX₂C motif (a conserved domain of the SAM enzyme superfamily) based on the Pfam database (PF04055) (58).

Sequences with amino acid identity greater than 80% (40) were classified as the same variant and validated using phylogenetic analysis. All putative *cfr*-like sequences were aligned using MAFFT v7.525 (59) with the L-INS-i algorithm. The resulting multiple sequence alignment was subsequently trimmed using TrimAl v1.5.rev1 (60) to remove ambiguously aligned regions. Phylogenetic relationships among all putative *cfr*-like sequences were then inferred using the maximum-likelihood method implemented in IQ-TREE v3.0.1 (61, 62), with the best-fit substitution model automatically selected using ModelFinder Plus (63–65).

### Screening, assembly, and annotation of *cfr*-positive genomes

For unassembled WGS data, to detect data harboring the *cfr* gene, a reference sequence index for the *cfr* gene and its homologous sequences was first constructed using BWA v0.7.18-r1243-dirty (66). The obtained WGS data were then aligned to this index. Subsequently, the SAM file of the alignment results was converted to BAM format using Samtools v1.20 (67), followed by sorting. Bedtools v2.31.1 (68) was used to calculate the genome coverage, and only those data sets with an average coverage greater than 5 for at least one reference sequence were retained. The obtained reads were trimmed by Trimmomatic v0.39.2 (69), and strain assemblies were generated using Unicycler v0.5.1 (70). For assembled genomes, BLAST was employed to screen for *cfr*-positive strains, with screening thresholds set at E-value <10⁻⁵, sequence identity >50%, and coverage >70%. Only genomes with an N50 ≥10,000 bp were retained for subsequent analysis.

The assembled genomes were annotated using PROKKA v1.14.6 (71). ARGs were identified using CARD (11) (last updated on 4 November 2023).

### *cfr* localization and mobile genetic element identification

To investigate the transmission mechanisms of the *cfr* gene, this study extracted the *cfr* gene along with its flanking 5,000 bp upstream and downstream sequences. The extraction script has been submitted to GitHub (https://github.com/cpu-helab/A-Systematic-Genomic-Framework-for-Tracking-Antibiotic-Resistance-Genes). The genomic localization (chromosome or plasmid) was determined using PlasFlow v1.1 (72).

To systematically investigate the dissemination mechanisms of the *cfr* gene across bacterial genera, this study conducted a comprehensive analysis of the distribution patterns of mobile genetic elements using the mobileOG-db Beatrix 1.6 (73).

GC content and GC skew were calculated to assess sequence composition and strand bias, serving as indicators for horizontal gene transfer (HGT). For cluster analysis, multi-dimensional features were standardized and grouped via K-means, with the optimal cluster number determined by the elbow method. Potential HGT events were identified by detecting outliers (±2 standard deviations) in both GC content and GC skew. Finally, predicted genes were categorized to explore the genomic context and evolutionary specialization of different *cfr* variants.

### Multilocus sequence typing of key strains

The multilocus sequence type (MLST) of the assembled genomes was determined using mlst v2.23.0 (https://github.com/tseemann/mlst) (74, 75). Core genome MLST allelic profiles of the genomes were constructed using PHYLOViZ v2.0 (76) (https://www.phyloviz.net/).

### Data visualization

Statistical analysis and data visualization were performed using R v4.2.0 with the ggplot2 v3.4.0 package for generating bar charts, scatter plots, box plots, and geographic distribution maps, and the pheatmap v1.0.12 package for generating heatmaps. Python language v3.9 was used for data processing and partial visualization, employing matplotlib v3.5.0 for basic graphics, seaborn v0.11.2 for statistical graphics enhancement, pandas v1.4.0 for data processing and integration, and NumPy v1.21.0 for numerical computation. Network diagrams and phylogenetic trees (77) were generated using Chiplot (https://www.chiplot.online). For figures requiring the combination of multiple subplots, Adobe Illustrator 2023 was used for layout and annotation to ensure graphical clarity, esthetics, and consistent font usage.

## SUPPLEMENTAL MATERIAL

Supplemental material aac.00122-26-s0001.docx Table S1; Fig. S1.

ASM does not own the copyrights to Supplemental Material that may be linked to, or accessed through, an article. The authors have granted ASM a non-exclusive, world-wide license to publish the Supplemental Material files. Please contact the corresponding author directly for reuse.

## References

[1] Gupta SK, Sharma P, McMillan EA, Jackson CR, Hiott LM, Woodley T, Humayoun SB, Barrett JB, Frye JG, McClelland M. 2019. Genomic comparison of diverse Salmonella serovars isolated from swine. PLoS One 14:e0224518. doi:10.1371/journal.pone.0224518 31675365 PMC6824618

[2] Nuanmuang N, Leekitcharoenphon P, Njage PMK, Gmeiner A, Aarestrup FM. 2023. An overview of antimicrobial resistance profiles of publicly available Salmonella genomes with sufficient quality and metadata. Foodborne Pathog Dis 20:405–413. doi:10.1089/fpd.2022.0080 37540138 PMC10510693

[3] Aarestrup FM, Brown EW, Detter C, Gerner-Smidt P, Gilmour MW, Harmsen D, Hendriksen RS, Hewson R, Heymann DL, Johansson K, Ijaz K, Keim PS, Koopmans M, Kroneman A, Lo Fo Wong D, Lund O, Palm D, Sawanpanyalert P, Sobel J, Schlundt J. 2012. Integrating genome-based informatics to modernize global disease monitoring, information sharing, and response. Emerg Infect Dis 18:e1. doi:10.3201/eid/1811.120453 PMC3559169 23092707

[4] Köser CU, Ellington MJ, Cartwright EJP, Gillespie SH, Brown NM, Farrington M, Holden MTG, Dougan G, Bentley SD, Parkhill J, Peacock SJ. 2012. Routine use of microbial whole genome sequencing in diagnostic and public health microbiology. PLoS Pathog 8:e1002824. doi:10.1371/journal.ppat.1002824 22876174 PMC3410874

[5] Rossen JWA, Friedrich AW, Moran-Gilad J, ESCMID Study Group for Genomic and Molecular Diagnostics (ESGMD). 2018. Practical issues in implementing whole-genome-sequencing in routine diagnostic microbiology. Clin Microbiol Infect 24:355–360. doi:10.1016/j.cmi.2017.11.001 29117578

[6] Djordjevic SP, Jarocki VM, Seemann T, Cummins ML, Watt AE, Drigo B, Wyrsch ER, Reid CJ, Donner E, Howden BP. 2024. Genomic surveillance for antimicrobial resistance - a One Health perspective. Nat Rev Genet 25:142–157. doi:10.1038/s41576-023-00649-y 37749210

[7] Yin X, Chen X, Jiang X-T, Yang Y, Li B, Shum MH-H, Lam TTY, Leung GM, Rose J, Sanchez-Cid C, et al.. 2023. Toward a universal unit for quantification of antibiotic resistance genes in environmental samples. Environ Sci Technol 57:9713–9721. doi:10.1021/acs.est.3c00159 37310875

[8] Chen X, Yin X, Xu X, Zhang T. 2025. Species-resolved profiling of antibiotic resistance genes in complex metagenomes through long-read overlapping with Argo. Nat Commun 16:1744. doi:10.1038/s41467-025-57088-y 39966439 PMC11836353

[9] Chiu CY, Miller SA. 2019. Clinical metagenomics. Nat Rev Genet 20:341–355. doi:10.1038/s41576-019-0113-7 30918369 PMC6858796

[10] Eyre DW. 2022. Infection prevention and control insights from a decade of pathogen whole-genome sequencing. J Hosp Infect 122:180–186. doi:10.1016/j.jhin.2022.01.024 35157991 PMC8837474

[11] Alcock BP, Huynh W, Chalil R, Smith KW, Raphenya AR, Wlodarski MA, Edalatmand A, Petkau A, Syed SA, Tsang KK, et al.. 2023. CARD 2023: expanded curation, support for machine learning, and resistome prediction at the comprehensive antibiotic resistance database. Nucleic Acids Res 51:D690–D699. doi:10.1093/nar/gkac920 36263822 PMC9825576

[12] Liu B, Pop M. 2009. ARDB--antibiotic resistance genes database. Nucleic Acids Res 37:D443–7. doi:10.1093/nar/gkn656 18832362 PMC2686595

[13] Tian C, Tang Z, Zhang X, Yao X, Li Y, Zhuang D, Luo Y, Li T, Bai L, Zhao F, Zhu L, Shi G, Jiang P, Gong Q, Zhou H, Gao H, Wu Q, Sang J, Liu X, Li X, Yu L, Zhang Z. 2025. Uncovering the gut microbiome and antibiotic resistome of mammals on the Tibetan Plateau. Sci China Life Sci 68:3646–3663. doi:10.1007/s11427-025-3047-5 40974529

[14] Jiang MZ, Liu C, Xu C, Jiang H, Wang Y, Liu SJ. 2024. Gut microbial interactions based on network construction and bacterial pairwise cultivation. Sci China Life Sci 67:1751–1762. doi:10.1007/s11427-023-2537-0 38600293

[15] Blake KS, Choi J, Dantas G. 2021. Approaches for characterizing and tracking hospital-associated multidrug-resistant bacteria. Cell Mol Life Sci 78:2585–2606. doi:10.1007/s00018-020-03717-2 33582841 PMC8005480

[16] Pal C, Bengtsson-Palme J, Kristiansson E, Larsson DGJ. 2016. The structure and diversity of human, animal and environmental resistomes. Microbiome 4:54. doi:10.1186/s40168-016-0199-5 27717408 PMC5055678

[17] Huijbers PMC, Flach CF, Larsson DGJ. 2019. A conceptual framework for the environmental surveillance of antibiotics and antibiotic resistance. Environ Int 130:104880. doi:10.1016/j.envint.2019.05.074 31220750

[18] Fu Y, Lu Y, Yang J, Liu H. 2025. Bacterial population dynamics and biosafety assessment in a closed bioregenerative life support system during the “Lunar palace 365” experiment. Sci China Life Sci 68:2137–2149. doi:10.1007/s11427-024-2857-1 40555951

[19] Peer MA, Nasir RA, Kakru DK, Fomda BA, Bashir G, Sheikh IA. 2011. Sepsis due to linezolid resistant Staphylococcus cohnii and Staphylococcus kloosii: first reports of linezolid resistance in coagulase negative staphylococci from India. Indian J Med Microbiol 29:60–62. doi:10.4103/0255-0857.76527 21304198

[20] Bassetti M, Baguneid M, Bouza E, Dryden M, Nathwani D, Wilcox M. 2014. European perspective and update on the management of complicated skin and soft tissue infections due to methicillin-resistant Staphylococcus aureus after more than 10 years of experience with linezolid. Clin Microbiol Infect 20 Suppl 4:3–18. doi:10.1111/1469-0691.12463 24580738

[21] Long KS, Vester B. 2012. Resistance to linezolid caused by modifications at its binding site on the ribosome. Antimicrob Agents Chemother 56:603–612. doi:10.1128/AAC.05702-11 22143525 PMC3264260

[22] Mendes RE, Deshpande LM, Jones RN. 2014. Linezolid update: stable in vitro activity following more than a decade of clinical use and summary of associated resistance mechanisms. Drug Resist Updat 17:1–12. doi:10.1016/j.drup.2014.04.002 24880801

[23] Bender J, Strommenger B, Steglich M, Zimmermann O, Fenner I, Lensing C, Dagwadordsch U, Kekulé AS, Werner G, Layer F. 2015. Linezolid resistance in clinical isolates of Staphylococcus epidermidis from German hospitals and characterization of two cfr-carrying plasmids. J Antimicrob Chemother 70:1630–1638. doi:10.1093/jac/dkv025 25740949

[24] Cuny C, Arnold P, Hermes J, Eckmanns T, Mehraj J, Schoenfelder S, Ziebuhr W, Zhao Q, Wang Y, Feßler AT, Krause G, Schwarz S, Witte W. 2017. Occurrence of cfr-mediated multiresistance in staphylococci from veal calves and pigs, from humans at the corresponding farms, and from veterinarians and their family members. Vet Microbiol 200:88–94. doi:10.1016/j.vetmic.2016.04.002 27102205

[25] Liu XQ, Wang J, Li W, Zhao LQ, Lu Y, Liu JH, Zeng ZL. 2017. Distribution of cfr in Staphylococcus spp. and Escherichia coli strains from pig farms in China and characterization of a novel cfr-carrying F43:A-:B- plasmid. Front Microbiol 8:329. doi:10.3389/fmicb.2017.00329 28293235 PMC5329041

[26] Shi Y, Li Y, Li H, Haerheng A, Marcelino VR, Lu M, Lemey P, Tang J, Bi Y, Pettersson JH-O, Bohlin J, Klaps J, Wu Z, Wan W, Sun B, Kang M, Holmes EC, He N, Su S. 2025. Extensive cross-species transmission of pathogens and antibiotic resistance genes in mammals neglected by public health surveillance. Cell 188:6591–6605. doi:10.1016/j.cell.2025.08.016 40865528

[27] Kehrenberg C, Schwarz S. 2006. Distribution of florfenicol resistance genes fexA and cfr among chloramphenicol-resistant Staphylococcus isolates. Antimicrob Agents Chemother 50:1156–1163. doi:10.1128/AAC.50.4.1156-1163.2006 16569824 PMC1426988

[28] Kehrenberg C, Aarestrup FM, Schwarz S. 2007. IS21-558 insertion sequences are involved in the mobility of the multiresistance gene cfr. Antimicrob Agents Chemother 51:483–487. doi:10.1128/AAC.01340-06 17145796 PMC1797725

[29] Wang Y, Zhang W, Wang J, Wu C, Shen Z, Fu X, Yan Y, Zhang Q, Schwarz S, Shen J. 2012. Distribution of the multidrug resistance gene cfr in Staphylococcus species isolates from swine farms in China. Antimicrob Agents Chemother 56:1485–1490. doi:10.1128/AAC.05827-11 22183168 PMC3294914

[30] Schwarz S, Werckenthin C, Kehrenberg C. 2000. Identification of a plasmid-borne chloramphenicol-florfenicol resistance gene in Staphylococcus sciuri. Antimicrob Agents Chemother 44:2530–2533. doi:10.1128/AAC.44.9.2530-2533.2000 10952608 PMC90098

[31] Kehrenberg C, Schwarz S, Jacobsen L, Hansen LH, Vester B. 2005. A new mechanism for chloramphenicol, florfenicol and clindamycin resistance: methylation of 23S ribosomal RNA at A2503. Mol Microbiol 57:1064–1073. doi:10.1111/j.1365-2958.2005.04754.x 16091044

[32] Long KS, Poehlsgaard J, Kehrenberg C, Schwarz S, Vester B. 2006. The Cfr rRNA methyltransferase confers resistance to phenicols, lincosamides, oxazolidinones, pleuromutilins, and streptogramin a antibiotics. Antimicrob Agents Chemother 50:2500–2505. doi:10.1128/AAC.00131-06 16801432 PMC1489768

[33] Smith LK, Mankin AS. 2008. Transcriptional and translational control of the mlr operon, which confers resistance to seven classes of protein synthesis inhibitors. Antimicrob Agents Chemother 52:1703–1712. doi:10.1128/AAC.01583-07 18299405 PMC2346656

[34] Polikanov YS, Starosta AL, Juette MF, Altman RB, Terry DS, Lu W, Burnett BJ, Dinos G, Reynolds KA, Blanchard SC, Steitz TA, Wilson DN. 2015. Distinct tRNA accommodation intermediates observed on the ribosome with the antibiotics hygromycin A and A201A. Mol Cell 58:832–844. doi:10.1016/j.molcel.2015.04.014 26028538 PMC4458074

[35] Nguyen LTT, Nguyen KNT, Le PNTA, Cafini F, Pascoe B, Sheppard SK, Nguyen TB, Nguyen TPH, Nguyen TV, Pham TTK, Morikawa K, Nguyen DQ, Duong HX. 2020. The emergence of plasmid-borne cfr-mediated linezolid resistant-staphylococci in Vietnam. J Glob Antimicrob Resist 22:462–465. doi:10.1016/j.jgar.2020.04.008 32348904

[36] Hansen LH, Planellas MH, Long KS, Vester B. 2012. The order Bacillales hosts functional homologs of the worrisome cfr antibiotic resistance gene. Antimicrob Agents Chemother 56:3563–3567. doi:10.1128/AAC.00673-12 22547628 PMC3393444

[37] Deshpande LM, Ashcraft DS, Kahn HP, Pankey G, Jones RN, Farrell DJ, Mendes RE. 2015. Detection of a new cfr-like gene, cfr(B), in Enterococcus faecium isolates recovered from human specimens in the United States as part of the sentry antimicrobial surveillance program. Antimicrob Agents Chemother 59:6256–6261. doi:10.1128/AAC.01473-15 26248384 PMC4576063

[38] Candela T, Marvaud JC, Nguyen TK, Lambert T. 2017. A cfr-like gene cfr(C) conferring linezolid resistance is common in Clostridium difficile. Int J Antimicrob Agents 50:496–500. doi:10.1016/j.ijantimicag.2017.03.013 28663118

[39] Guerin F, Sassi M, Dejoies L, Zouari A, Schutz S, Potrel S, Auzou M, Collet A, Lecointe D, Auger G, Cattoir V. 2020. Molecular and functional analysis of the novel cfr(D) linezolid resistance gene identified in Enterococcus faecium. J Antimicrob Chemother 75:1699–1703. doi:10.1093/jac/dkaa125 32277823

[40] Stojković V, Ulate MF, Hidalgo-Villeda F. 2019. cfr(B), cfr(C), and a New cfr-Like Gene, cfr(E), in Clostridium difficile strains recovered across Latin America. Antimicrob Agents Chemother 64. doi:10.1128/AAC.01074-19 PMC7187602 31685464

[41] Atkinson GC, Hansen LH, Tenson T, Rasmussen A, Kirpekar F, Vester B. 2013. Distinction between the Cfr methyltransferase conferring antibiotic resistance and the housekeeping RlmN methyltransferase. Antimicrob Agents Chemother 57:4019–4026. doi:10.1128/AAC.00448-13 23752511 PMC3719738

[42] Naghavi M, Vollset SE, Ikuta KS, Swetschinski LR, Gray AP, Wool EE, Robles Aguilar G, Mestrovic T, Smith G, Han C, et al.. 2024. Global burden of bacterial antimicrobial resistance 1990–2021: a systematic analysis with forecasts to 2050. The Lancet 404:1199–1226. doi:10.1016/S0140-6736(24)01867-1 PMC11718157 39299261

[43] Sati H, Carrara E, Savoldi A, Hansen P, Garlasco J, Campagnaro E, Boccia S, Castillo-Polo JA, Magrini E, Garcia-Vello P, Wool E, Gigante V, Duffy E, Cassini A, Huttner B, Pardo PR, Naghavi M, Mirzayev F, Zignol M, Cameron A, Tacconelli E, WHO Bacterial Priority Pathogens List Advisory Group. 2025. The WHO bacterial priority pathogens list 2024: a prioritisation study to guide research, development, and public health strategies against antimicrobial resistance. Lancet Infect Dis 25:1033–1043. doi:10.1016/S1473-3099(25)00118-5 40245910 PMC12367593

[44] Kardos G, Laczkó L, Kaszab E, Timmer B, Szarka K, Prépost E, Bányai K. 2024. Phylogeny of transferable oxazolidinone resistance genes and homologs. Antibiotics (Basel) 13:311. doi:10.3390/antibiotics13040311 38666987 PMC11047308

[45] Rotta C, Poehlein A, Schwarz K, McClure P, Daniel R, Minton NP. 2015. Closed genome sequence of Clostridium pasteurianum ATCC 6013. Genome Announc 3:e01596-14. doi:10.1128/genomeA.01596-14 25700419 PMC4335343

[46] Wang R, Peng C, Zhao H, Liu H, Wei T, Wang L, Li H. 2025. Strengthening mechanisms of indigenous bacteria in granite residual soil improvement via microbial induced calcite precipitation. Sci Rep 16:2782. doi:10.1038/s41598-025-32718-z 41398055 PMC12823652

[47] Xia X, Lv T, Zheng L, Zhao Y, Shen P, Zhu D, Chen Y. 2024. Genomic epidemiology of Clostridioides difficile ST81 in multiple hospitals in China. Infect Drug Resist 17:5535–5544. doi:10.2147/IDR.S492668 39676847 PMC11646370

[48] Lee JB, Lim JH, Park JH, Lee GY, Park KT, Yang SJ. 2024. Genetic characteristics and antimicrobial resistance of Staphylococcus aureus isolates from pig farms in Korea: emergence of cfr-positive CC398 lineage. BMC Vet Res 20:503. doi:10.1186/s12917-024-04360-w 39487420 PMC11529005

[49] Ngbede EO, Sy I, Akwuobu CA, Nanven MA, Adikwu AA, Abba PO, Adah MI, Becker SL. 2023. Carriage of linezolid-resistant enterococci (LRE) among humans and animals in Nigeria: coexistence of the cfr, optrA, and poxtA genes in Enterococcus faecium of animal origin. J Glob Antimicrob Resist 34:234–239. doi:10.1016/j.jgar.2023.07.016 37516354

[50] Fioriti S, Morroni G, Coccitto SN, Brenciani A, Antonelli A, Di Pilato V, Baccani I, Pollini S, Cucco L, Morelli A, Paniccià M, Magistrali CF, Rossolini GM, Giovanetti E. 2020. Detection of oxazolidinone resistance genes and characterization of genetic environments in Enterococci of Swine Origin, Italy. Microorganisms 8:2021. doi:10.3390/microorganisms8122021 33348682 PMC7766396

[51] Ruiz-Ripa L, Feßler AT, Hanke D, Eichhorn I, Azcona-Gutiérrez JM, Pérez-Moreno MO, Seral C, Aspiroz C, Alonso CA, Torres L, Alós JI, Schwarz S, Torres C. 2020. Mechanisms of linezolid resistance among Enterococci of clinical origin in spain—detection of optrA- and cfr(D)-carrying E. faecalis. Microorganisms 8:1155. doi:10.3390/microorganisms8081155 32751552 PMC7464793

[52] Nasir SAR, Zeeshan M, Ghanchi N, Saeed N, Ghayas H, Zaka S, Ashraf J, Jabeen K, Farooqi J, Hasan Z, Fatima T, Rezwan F, Mahmood SF, Arshad M, Khan E, Ozer EA, Hasan R. 2024. Linezolid-resistant Enterococcus faecium clinical isolates from Pakistan: a genomic analysis. BMC Microbiol 24:347. doi:10.1186/s12866-024-03491-2 39277715 PMC11401331

[53] Cinthi M, Coccitto SN, Simoni S, Gherardi G, Palamara AT, Di Lodovico S, Di Giulio M, Du X-D, Vignaroli C, Brenciani A, Giovanetti E. 2025. The optrA, cfr(D) and vanA genes are co-located on linear plasmids in linezolid- and vancomycin-resistant enterococcal clinical isolates in Italy. J Antimicrob Chemother 80:1362–1370. doi:10.1093/jac/dkaf082 40094923

[54] Altschul SF, Gish W, Miller W, Myers EW, Lipman DJ. 1990. Basic local alignment search tool. J Mol Biol 215:403–410. doi:10.1016/S0022-2836(05)80360-2 2231712

[55] Altschul SF, Madden TL, Schäffer AA, Zhang J, Zhang Z, Miller W, Lipman DJ. 1997. Gapped BLAST and PSI-BLAST: a new generation of protein database search programs. Nucleic Acids Res 25:3389–3402. doi:10.1093/nar/25.17.3389 9254694 PMC146917

[56] Buchfink B, Xie C, Huson DH. 2015. Fast and sensitive protein alignment using DIAMOND. Nat Methods 12:59–60. doi:10.1038/nmeth.3176 25402007

[57] Eddy SR. 2011. Accelerated profile HMM searches. PLoS Comput Biol 7:e1002195. doi:10.1371/journal.pcbi.1002195 22039361 PMC3197634

[58] Paysan-Lafosse T, Andreeva A, Blum M, Chuguransky SR, Grego T, Pinto BL, Salazar GA, Bileschi ML, Llinares-López F, Meng-Papaxanthos L, Colwell LJ, Grishin NV, Schaeffer RD, Clementel D, Tosatto SCE, Sonnhammer E, Wood V, Bateman A. 2025. The Pfam protein families database: embracing AI/ML. Nucleic Acids Res 53:D523–D534. doi:10.1093/nar/gkae997 39540428 PMC11701544

[59] Katoh K, Standley DM. 2013. MAFFT multiple sequence alignment software version 7: improvements in performance and usability. Mol Biol Evol 30:772–780. doi:10.1093/molbev/mst010 23329690 PMC3603318

[60] Capella-Gutiérrez S, Silla-Martínez JM, Gabaldón T. 2009. trimAl: a tool for automated alignment trimming in large-scale phylogenetic analyses. Bioinformatics 25:1972–1973. doi:10.1093/bioinformatics/btp348 19505945 PMC2712344

[61] Nguyen L-T, Schmidt HA, von Haeseler A, Minh BQ. 2015. IQ-TREE: a fast and effective stochastic algorithm for estimating maximum-likelihood phylogenies. Mol Biol Evol 32:268–274. doi:10.1093/molbev/msu300 25371430 PMC4271533

[62] Minh BQ, Schmidt HA, Chernomor O, Schrempf D, Woodhams MD, von Haeseler A, Lanfear R. 2020. IQ-TREE 2: new models and efficient methods for phylogenetic inference in the genomic era. Mol Biol Evol 37:1530–1534. doi:10.1093/molbev/msaa015 32011700 PMC7182206

[63] Kalyaanamoorthy S, Minh BQ, Wong TKF, von Haeseler A, Jermiin LS. 2017. ModelFinder: fast model selection for accurate phylogenetic estimates. Nat Methods 14:587–589. doi:10.1038/nmeth.4285 28481363 PMC5453245

[64] He WT, Ji X, He W, Dellicour S, Wang S, Li G, Zhang L, Gilbert M, Zhu H, Xing G, Veit M, Huang Z, Han GZ, Huang Y, Suchard MA, Baele G, Lemey P, Su S. 2020. Genomic epidemiology, evolution, and transmission dynamics of porcine deltacoronavirus. Mol Biol Evol 37:2641–2654. doi:10.1093/molbev/msaa117 32407507 PMC7454817

[65] He WT, Bollen N, Xu Y, Zhao J, Dellicour S, Yan Z, Gong W, Zhang C, Zhang L, Lu M, Lai A, Suchard MA, Ji X, Tu C, Lemey P, Baele G, Su S. 2022. Phylogeography reveals association between swine trade and the spread of porcine epidemic diarrhea virus in China and across the World. Mol Biol Evol 39:msab364. doi:10.1093/molbev/msab364 34951645 PMC8826572

[66] Li H, Durbin R. 2009. Fast and accurate short read alignment with Burrows–Wheeler transform. Bioinformatics 25:1754–1760. doi:10.1093/bioinformatics/btp324 19451168 PMC2705234

[67] Li Heng, Handsaker B, Wysoker A, Fennell T, Ruan J, Homer N, Marth G, Abecasis G, Durbin R, 1000 Genome Project Data Processing Subgroup. 2009. The sequence alignment/map format and SAMtools. Bioinformatics 25:2078–2079. doi:10.1093/bioinformatics/btp352 19505943 PMC2723002

[68] Quinlan AR, Hall IM. 2010. BEDTools: a flexible suite of utilities for comparing genomic features. Bioinformatics 26:841–842. doi:10.1093/bioinformatics/btq033 20110278 PMC2832824

[69] Bolger AM, Lohse M, Usadel B. 2014. Trimmomatic: a flexible trimmer for Illumina sequence data. Bioinformatics 30:2114–2120. doi:10.1093/bioinformatics/btu170 24695404 PMC4103590

[70] Wick RR, Judd LM, Gorrie CL, Holt KE. 2017. Unicycler: resolving bacterial genome assemblies from short and long sequencing reads. PLoS Comput Biol 13:e1005595. doi:10.1371/journal.pcbi.1005595 28594827 PMC5481147

[71] Seemann T. 2014. Prokka: rapid prokaryotic genome annotation. Bioinformatics 30:2068–2069. doi:10.1093/bioinformatics/btu153 24642063

[72] Krawczyk PS, Lipinski L, Dziembowski A. 2018. PlasFlow: predicting plasmid sequences in metagenomic data using genome signatures. Nucleic Acids Res 46:e35. doi:10.1093/nar/gkx1321 29346586 PMC5887522

[73] Brown CL, Mullet J, Hindi F, Stoll JE, Gupta S, Choi M, Keenum I, Vikesland P, Pruden A, Zhang L. 2022. mobileOG-db: a manually curated database of protein families mediating the life cycle of bacterial mobile genetic elements. Appl Environ Microbiol 88:e0099122. doi:10.1128/aem.00991-22 36036594 PMC9499024

[74] Jolley Keith A, Maiden MCJ. 2010. BIGSdb: Scalable analysis of bacterial genome variation at the population level. BMC Bioinformatics 11:595. doi:10.1186/1471-2105-11-595 21143983 PMC3004885

[75] Jolley K.A, Bray JE, Maiden MCJ. 2018. Open-access bacterial population genomics: BIGSdb software, the PubMLST.org website and their applications. Wellcome Open Res 3:124. doi:10.12688/wellcomeopenres.14826.1 30345391 PMC6192448

[76] Ribeiro-Gonçalves B, Francisco AP, Vaz C, Ramirez M, Carriço JA. 2016. PHYLOViZ Online: web-based tool for visualization, phylogenetic inference, analysis and sharing of minimum spanning trees. Nucleic Acids Res 44:W246–51. doi:10.1093/nar/gkw359 27131357 PMC4987911

[77] Xie J, Chen Y, Cai G, Cai R, Hu Z, Wang H. 2023. Tree visualization by one table (tvBOT): a web application for visualizing, modifying and annotating phylogenetic trees. Nucleic Acids Res 51:W587–W592. doi:10.1093/nar/gkad359 37144476 PMC10320113

