# Supplementary material for: A genomic framework for tracking antibiotic resistance genes: global dissemination of cfr-family genes
Source: Antimicrob Agents Chemother. 2026 Jul 10;70(8):e00122-26. doi: 10.1128/aac.00122-26 (PMC13436266; doi:10.1128/aac.00122-26)
Supplement: Supplementary file 1 [file aac.00122-26-s0001.docx]

**Supplementary File.**
**Table S1. Reference sequence standards for cfr variant classification.**

| ***cfr* Type** | **DNA ID** | **AA ID** |
| --- | --- | --- |
| *cfr*(A) | AM408573.1 | CAL64019.1 |
| *clbA* | CP006845.1 | AGZ55247.1 |
| *clbC* | AP006627.1 | BAD63613.1 |
| *cfr*(B) | HG002396.1 | CDF47262.1 |
| *clbB* | AP008955.1 | BAH45481.1 |
| *cipA* | CP001793.1 | ACX65640.1 |
| *cfr*(C) | - | CCL89685 |
| *cfr*(D) | MG707078.1 | AXY65402.1 |
| *cfr*(E) | QUDP01000003.1 | RHO92765.1 |
| *cfr*(G) | AM412317.1 | CAL84423.1 |

**Figure S1. Evidence of Horizontal Transfer Based on GC Anomalies.**

Figure S1. Evidence of Horizontal Transfer Based on GC Anomalies.

A. A normal distribution plot of GC content. The vertical dashed lines mark the statistical threshold of ±2σ. Regions highlighted in red represent sequence areas identified as having significantly abnormal GC content.

B. A normal distribution plot of GC deviation. The vertical dashed lines mark the statistical threshold of ±2σ. Regions highlighted in blue represent sequence areas identified as having significantly abnormal GC deviation.
